# Predicting Depression with Psychopathology and Temperament Traits: The Northern Finland 1966 Birth Cohort

**DOI:** 10.1155/2012/160905

**Published:** 2012-08-16

**Authors:** Jouko Miettunen, Matti Isohanni, Tiina Paunio, Nelson Freimer, Anja Taanila, Jesper Ekelund, Marjo-Riitta Järvelin, Matti Joukamaa, Dirk Lichtermann, Heli Koivumaa-Honkanen, Juha Veijola

**Affiliations:** ^1^Department of Psychiatry, Institute of Clinical Medicine, University of Oulu, P.O. Box 5000, 90014 Oulu, Finland; ^2^Clinic of Psychiatry, Oulu University Hospital, Oulu, Finland; ^3^Department of Chronic Disease Prevention, National Institute for Health and Welfare, Haartmaninkatu 8, 00251 Helsinki, Finland; ^4^UCLA Center for Neurobehavioral Genetics, Semel Institute for Neuroscience and Human Behavior, Gonda Building, Room 3506, 695 Charles E. Young Drive South, Los Angeles, CA 90095, USA; ^5^Institute of Health Sciences, University of Oulu, P.O. Box 5000, 90014 Oulu, Finland; ^6^Unit of General Practice, Oulu University Hospital, 90029 Oulu, Finland; ^7^Department of Mental Health and Substance Use Services, National Institute for Health and Welfare, P.O. Box 30, 00271 Helsinki, Finland; ^8^Department of Epidemiology and Biostatistics, Imperial College London, London SW7 2AZ, UK; ^9^Social Psychiatry Unit, School of Health Sciences, University of Tampere, 33014 Tampere, Finland; ^10^Department of Psychiatry, Tampere University Hospital, Tampere, Finland; ^11^Methadone Maintenance Clinic “Café Ersatz”, Münsterstraße 18, 53111 Bonn, Germany; ^12^Department of Psychiatry, University of Eastern Finland and Kuopio University Hospital, P.O. Box 1777, 70211 Kuopio, Finland; ^13^Department of Psychiatry, South-Savonia Hospital District, Mikkeli, Finland; ^14^Department of Psychiatry, North Karelia Central Hospital, Joensuu, Finland; ^15^Department of Psychiatry, SOSTERI, Savonlinna, Finland; ^16^Department of Psychiatry, SOTE, Iisalmi, Finland; ^17^Department of Psychiatry, Lapland Hospital District, Rovaniemi, Finland

## Abstract

We studied the concurrent, predictive, and discriminate validity of psychopathology scales (e.g., schizotypal and depressive) and temperament traits for hospitalisations due to major depression. Temperament, perceptual aberration, physical and social anhedonia, Depression Subscale of Symptom Checklist (SCL-D), Hypomanic Personality Scale, Schizoidia Scale, and Bipolar II Scale were completed as part of the 31-year follow-up survey of the prospective Northern Finland 1966 Birth Cohort (*n* = 4941; 2214 males). Several of the scales were related to depression. Concurrent depression was especially related to higher perceptual aberration (effect size when compared to controls, *d* = 1.29), subsequent depression to high scores in SCL-D (*d* = 0.48). Physical anhedonia was lower in subjects with subsequent depression than those with other psychiatric disorders (*d* = −0.33, nonsignificant). Participants with concurrent (*d* = 0.70) and subsequent (*d* = 0.54) depression had high harm avoidance compared to controls, while differences compared to other psychiatric patients were small. Subjects with depression differed from healthy controls in most of the scales. Many of the scales were useful predictors for future hospital treatments, but were not diagnosis-specific. High harm avoidance is a potential indicator for subsequent depression.

## 1. Introduction

Several studies have attempted to predict psychiatric symptoms or disorders using various psychological and psychiatric instruments [1, 2]. These scales have clinical importance, for example, when identifying high-risk individuals. They may also help to detect intermediate phenotypes for psychiatric disorders [3]. Potential psychopathological intermediate phenotypes of depression include, for example, depressed mood and anhedonia [4]. The objectiveness of these scales adds to their usefulness in etiological research of psychiatric disorders [5].

Numerous instruments have been used to evaluate depressive symptoms in the general population. For instance, the Symptom Checklist was developed to screen for depression and anxiety in the general population [6]. Personality traits, such as those measured with Cloninger's [7] temperament dimensions, have been associated with several psychiatric disorders [8]. Particularly high harm avoidance has been associated with depression [8]. The previous longitudinal studies in relation to depression are very few and have included relatively short follow-up period [9, 10]. These studies indicate that harm avoidance may be a phenotypic indicator for risk of depressive episodes [10]. Schizotypal traits, such as anhedonia, are key features both in schizophrenia and depression. In patients with schizophrenia, anhedonia has been strongly associated with the depressive syndrome [11]. 

The aim of the present study was to evaluate the concurrent, predictive, and discriminate validity of several psychological scales for depression, using a large representative population based sample of adults, with adequate followup. Our hypothesis was that subjects with either concurrent or subsequent depression would score differently in schizotypal and depressive symptom scales and temperament dimensions, when compared to subjects with other psychiatric disorders or healthy controls.

## 2. Material and Methods

### 2.1. Participants

The Northern Finland 1966 Birth Cohort (NFBC 1966) is an unselected, general population-based birth cohort ascertained during mid-pregnancy. It comprised of 12,058 live-born children in the Finnish provinces of Lapland and Oulu [12]. We required that all subjects had been living in Finland at the age of 16 (*n* = 10,933; 5,589 males and 5,344 females), as we have previously validated psychiatric diagnoses from the Finnish Hospital Discharge Register in this subsample [13, 14]. Permission to gather data was obtained from the Ministry of Social and Health Affairs and the study design has been approved by, and is under review of, the Ethical Committee of The Northern Ostrobothnia Hospital District. After complete description of the study, written informed consent was obtained from study participants.

In 1997, the questionnaires used for the 31-year followup of the cohort included a large collection of psychopathology scales and four temperament dimensions that were given to all cohort members who participated in a clinical examination [15]. Participants completed these scales at home and returned them in the envelopes provided. The 12-item version of the Infrequency Scale [16] was used to assess careless responding. It contains items that are very unlikely to be true and identifies random response styles. Participants who endorsed three or more items (*n* = 105) on this scale were excluded from further analyses. The Symptom Checklist-25 [6, 17, 18] was sent, by post, in a different set of questions, together with several sociodemographic questions and an invitation letter for the clinical examination. In total, 4,941 participants (2,214 males and 2,727 females) adequately completed at least one of these scales.

The Finnish Hospital Discharge Register (FHDR) covers all mental and general hospitals as well as beds in local health centres, prison and military hospitals and private hospitals nationwide. All cohort members, over 16 years old, appearing on the FHDR until the end of 1997 for any mental disorder were identified and their diagnoses were rechecked twice by psychiatrists using DSM-III-R criteria [19]. The reliability of this procedure was moderate for depression *κ* = 0.57). A more detailed description of the validation process is presented elsewhere [13, 14].

Data was also collected from postal questionnaires sent to all subjects in 1977 and outpatient treatments with self-reported diagnoses. Subjects were asked whether they had any of the following: depression, psychosis, alcohol use disorder, other substance use disorder, or any other psychiatric disorder diagnosed by a medical doctor. Three groups were formed: “depressive disorder” (*n* = 204; 68 males, 33%), “other psychiatric disorders” (*n* = 150; 77 males, 51%), and “no psychiatric disorders” (*n* = 4587; 2069 males, 45%). The “depressive disorder” group included subjects with validated hospital care diagnoses of psychotic depression (DSM-III-R codes: 296.24 and 296.34; *n* = 2) or nonpsychotic depression (296.22, 296.23, 296.31, 296.33, and 311.00; *n* = 20), or self-reported depressions (*n* = 182). “Other psychiatric disorders” included subjects with other psychiatric diagnoses based on either hospital (*n* = 70) or self-reported data (*n* = 80). In the followup, the same groups were used, but only with nonvalidated data from the FHDR from 1998 to 2010, classified as described above. The distribution of the diagnoses for major depressive disorders (ICD-10 diagnoses: F32-F33 and F341) are presented in Section 3.

When comparing the final sample at the 31-year followup to subjects alive at the age of 16 years, females participated more commonly than males (51.4% versus 40.4%; *χ* ^2^ 134.81, *P* < 0.001) and those with tertiary (more than 12 years) and secondary level (10–12 years) education more commonly than those with basic level (9 or less years) education (49.5% and 49.7% versus 25.1%; *χ* ^2^ 343.73, degrees of freedom = 2, *P* < 0.001). The attrition analyses for the 31-year follow-up study have previously been described in detail. For individuals with psychiatric hospital diagnoses until 1997, the participation rate was 42% among those with mood disorders and 54% among controls [15].

### 2.2. Instruments

The questionnaire given to subjects consisted of mental health related true/false questions, which were collected from several psychopathology (e.g., schizotypal) scales and four temperament subscales. All these items, and the 12 items from the Infrequency Scale, were randomly rearranged into a 354-item questionnaire, called “Survey of Opinions and Experiences.” We used the temperament subscales (novelty seeking, harm avoidance, reward dependence, and persistence) from the Temperament and Character Inventory (TCI). The schizotypal scales were the following: Physical Anhedonia Scale (PAS), Social Anhedonia Scale (SAS), Perceptual Aberration Scale (PER), Hypomanic Personality Scale (HPS), Bipolar II Scale (BIP2), and Schizoidia Scale (SCHD). From a separate survey, we included the depression subscale (15 items) of Symptom Checklist (SCL-D) in order to also assess depressive symptoms. The answers to SCL are scored on a scale from 1 (not bothered) to 4 (extremely bothered) [6]. The references and short descriptions for the scales are presented in Table 1. Firstly, we studied these scales in respect to previous hospitalization or current self-reported psychiatric diagnosis in 1997. Secondly, among those without previous psychiatric diagnosis in 1997, these scales were studied in respect to subsequent hospitalizations due to psychiatric causes between 1998 and 2010.

The original English versions of the scales, except SCL-25, were translated into Finnish by one investigator and then back-translated blindly to the original English scale by a professional English translator. The original version and the back-translation were compared, and corrections were made accordingly. The translation was tested in a sample of 50 laboratory workers, and the results indicated that no questions required revision. We used an earlier Finnish translation of the SCL in the current study. We have previously presented validity results in this sample for the SCL-25 [18], temperament [26], and schizotypal scales [27].

### 2.3. Statistical Methods

We present means, standard deviations (SD), and Student's *t*-tests for continuous variables. Mean scores between different groups were compared using effect sizes (Cohen's *d*) and two-way analysis of variance, where diagnostic group and gender were used as grouping variables and scale scores as dependent variables. Gender was used as a covariate, as previous meta-analyses have found gender differences in these scales [28, 29]. Cohen [30] interpreted *d* values of 0.2 to 0.5 as small, 0.5 to 0.8 as medium, and 0.8 or over as large effects. We studied associations of all the scales both to depressions and to other psychiatric disorders in order to estimate the specificity of the scales. As substance use disorder is a common diagnosis in other psychiatric diagnoses and a common comorbid diagnosis in depression, we performed additional analysis with substance abuse as a covariate in analysis of variance. All tests are two-tailed. The data was analyzed using the IBM SPSS v. 20.

## 3. Results

Table 2 shows mean (SD) values for the different scales in categories of concurrent depression, other psychiatric disorders, and no psychiatric disorders. In 1997, those with depression scored statistically significantly (*P* < 0.05) higher than those without previous psychiatric disorders in all studied psychopathology scales, except PAS. The difference was highest in SCL-D (means 1.78 versus 1.33, *d* = 1.29) followed by perceptual aberration scale (means 5.09 versus 2.24, *d* = 0.90). In temperament dimensions, this difference was statistically significant only in TCI-HA, with higher scores among those with depression (*d* = 0.70). Almost all the scales were related to depression. However, it was only in SCL-D that those with depression differed statistically significantly from those with other psychiatric disorders with a small effect size (*d* = 0.20). No significant differences between these two groups were found in temperament dimensions (Table 2).

Among those with no psychiatric disorders until 1997, 51 (26 males, 51%) new cases with depression requiring hospitalizations emerged during the subsequent followup of thirteen years. This group included subjects with psychotic depression (F323, F333), *n* = 9 and nonpsychotic depression (F32-F33, not F323, F33; F341), *n* = 42. In this followup, 57 (36 males, 63%) individuals were hospitalized for other psychiatric disorders. This group included various psychiatric diagnoses. The most common diagnoses were substance use disorder (*n* = 30), nonaffective psychosis (*n* = 14), and anxiety disorders (*n* = 12).

Of the psychopathology scales, the bipolar II scale (*d* = 0.52), SCL-D (*d* = 0.48), SCHD (*d* = 0.46), and SAS (*d* = 0.45) were the strongest predictors for new cases of depression requiring hospitalization. However, the scales were not diagnosis-specific. The greatest difference between depression and other psychiatric disorders requiring hospitalization (*d* = −0.33; NS) was found in PAS.

In temperament dimensions, new cases of depression requiring hospitalization scored high in the harm avoidance when compared to those with no hospitalization due to psychiatric causes (16.96 versus 13.77; *P* < 0.001, *d* = 0.54). No significant differences were found between the two psychiatric groups. Novelty seeking had the highest effect size (ns, *d* = −0.32), with lower scores among those with hospitalization due to depression. In additional analyses, between those with depression and other psychiatric disorders we controlled for substance use disorders. The difference between these two groups was statistically significant in harm avoidance in concurrent analyses (*F* = 7.12, *P* = 0.008) with higher scores among those with hospitalization due to depression. Differences in other temperament traits or in predictive analyses were nonsignificant (Table 3).

## 4. Discussion

We studied the concurrent, predictive, and discriminate validity of several psychopathology scales and temperamental dimensions in relation to depression. Many of them were related to depression but also to other psychiatric disorders. Harm avoidance (*d* = 0.54) as a temperament dimension and the bipolar II scale (*d* = 0.52) best predicted depression, and also the depression subscale of the SCL associated with depression, especially concurrently (*d* = 1.29). These findings reflect the relatively high predictive value of the scales and confirm their utility as clinical tools for the identification of individuals at risk for psychiatric disorders. None of the scales discriminated well subjects with depression from subjects with other psychiatric disorders. However, when we took into account substance use disorders as a covariate in concurrent analyses, individuals with depression scored statistically significantly higher in harm avoidance than those with other psychiatric disorders.

### 4.1. Schizotypal Scales

We found that many of the psychopathology scales related to both concurrent and subsequent depression. However, these scales were not diagnosis- specific as these traits were also common in other psychiatric disorders. In the schizotypal (PAS, SAS, and PER) scales, there have been some previous case-control studies in depression, with small sample sizes. These studies have found large effect sizes between psychiatric cases and healthy controls (e.g., students), with cases scoring higher in all of them. In physical anhedonia, effect sizes have varied between 0.92 [31] and 1.79 [32], and in social anhedonia between 0.86 [33] and 1.19 [34]. In perceptual aberration, Katsanis et al. [33] found depressive patients scored higher than healthy controls, with an effect size of 0.57. Loas et al. [35] note that physical anhedonia in depressed patients seems to relate to the severity of the depression and does not appear to identify quantitatively distinct subgroup.

We have previously studied these schizotypal scales in schizophrenia [36]. Individuals with concurrent depression (*n* = 202) scored lower than those with schizophrenia (*n* = 29) in all schizotypal scales, for example, in physical anhedonia, mean score in schizophrenia was 18.38 and in depression 14.68. When predicting these disorders, differences in scores were smaller; however, those with subsequent schizophrenia scored substantially higher in perceptual aberration (means 4.30 versus 2.96) and hypomanic personality (17.10 versus 12.37) than those with subsequent depression. Interestingly, our results contradict some previous studies such as Blanchard et al. [34], who found that depressive patients had higher scores for social anhedonia than schizophrenia patients (means 18 versus 15). They concluded that schizophrenia patients seem to have stable high scores in social anhedonia whereas, in depressive patients, it correlates with the clinical state. These differences may relate to our sample being based on lifetime diagnoses in population, including also individuals without current treatment or symptoms.

### 4.2. Depressive Symptoms

Sandanger et al. [37] previously conducted a cross-sectional study of the specificity of SCL-25 for depression. They found that a cutoff of 1.75 points in average was a powerful predictor for depression (crude odds ratio, OR 14.0). However, the scale was also significantly associated with different anxiety disorders (ORs 2.2 to 4.4) and with any disorder (OR 3.9). In a previous study of the NFBC 1966, Veijola et al. [18] compared the SCL-25 with Structured Clinical Interview for DSM-III-R (SCID) diagnoses in a subsample of the cohort. They concluded that SCL-25 might be useful for screening purposes in primary health care and epidemiological surveys. They noted that cases with psychiatric comorbid disorders were screened successfully using the instrument. According to the present study, the depression subscale of the SCL was a quite good (*d* = 0.48) predictor of new cases of depression requiring hospitalization, when compared to those with no new psychiatric disorders requiring hospitalization during the followup. However, specificity was poor (*d* = 0.09), indicating that depressive symptoms also relate to other subsequent psychiatric disorders (Table 3).

### 4.3. Temperament Dimensions

We found a strong association between depression and harm avoidance, as has also been noted in a recent meta-analysis comparing different psychiatric disorders [8]. This meta-analysis also found a small negative effect size (*d* = −0.20) of lower novelty seeking in major depression, whereas the effect in harm avoidance was very large (*d* = 1.64) when compared to controls. High harm avoidance in depression has been shown to remain, after controlling for age, gender, diagnosis, and depressive state effects [38]. Harm avoidance has also been shown to decrease after treatment response [39]. Interestingly, never-depressed siblings of the probands with major depression are reported as having higher harm avoidance compared to never-depressed siblings of never-depressed controls. Thus, the authors suggested that harm avoidance might be a trait-like characteristic related to a familial vulnerability to depression [40].

In our sample, individuals with concurrent or previous depression had very similar temperament when compared to individuals with other psychiatric disorders, with all the effect sizes being below 0.20. In the extensive meta-analysis by Miettunen and Raevuori [8], depression (23 studies) had higher P, RD, and HA than schizophrenia, but quite similar temperament when compared to alcohol use disorders. It is worth mentioning that, in the present study, about half of the patients with subsequent other psychiatric disorder had a substance use diagnosis (as their only diagnosis or in addition to some other diagnosis). Because of this, we performed additional analyses substance use disorder as additional covariate. We found that in concurrent, but not in predictive analyses, individuals with depression scored significantly higher than those with other psychiatric disorders in harm avoidance. 

When we compared current results to our previous schizophrenia study with the NFBC 1966 [36], in those who have already received a diagnosis, harm avoidance was lower (*d* = −0.50) and novelty seeking higher (*d* = 0.48) in depression than in schizophrenia. Those with subsequent depression substantially had higher scores, especially in reward dependency, than those with subsequent schizophrenia (mean scores 14.2 versus 11.8; *d* = 0.65).

### 4.4. Strengths and Limitations

The sample was relatively large and population-based. Unlike many of the previous studies, we also included a comparison group with other psychiatric disorders. The extensive set of scales used is also an advantage. A limitation of the sample is that, although it was large, a low number of cases during the followup also meant that it was not possible to predict diagnoses more specifically. Due to cohort design, it only consists of one age group. As we used the nationwide hospital register to find new cases of depression, our outcome only indicates a very severe form of depression. We cannot avoid the possibility that, at the end of followup, high scorers without psychiatric diagnosis may have a depression diagnosis not requiring hospitalization, as most of the subjects with depressive episode are not treated in psychiatric hospital facilities [41]. It should also be noted that quite a large proportion of eligible subjects did not participate or gave inadequate answers [15].

## 5. Conclusions

In most of the scales, subjects with depression differed from healthy controls. Many of the scales were useful predictors for subsequent new hospitalisations due to depression. With lack of diagnosis specificity, these scales also predicted other psychiatric diagnoses. Thus, these scales are useful both in clinical work and in etiological research of psychiatric disorders.

## Figures and Tables

**Table 1:** Psychological scales used in the 31-year followup of the Northern Finland 1966 Birth Cohort.

Instrument (abbreviation)	Number of items	Description of high scorers	Reference(s)
Revised Physical Anhedonia Scale (PAS)	61	Lowered ability to experience physical and sensory pleasures	Chapman et al., [20]
Revised Social Anhedonia Scale (SAS)	40	Schizoid lack of interest in social interaction	Chapman et al., [20]; Eckbladet al., [21]
Perceptual Aberration Scale (PER)	35	Have distorted perception of own body and other objects	Chapman et al., [22]
Hypomanic Personality Scale (HPS)	48	Energetic, upbeat, gregarious people, often able to work long hours	Eckblad and Chapman, [23]
Bipolar II Scale (BIP2)	31	Designed to predict bipolar II disorder among unipolar subjects	Akiskal et al., [24]
Schizoidia Scale (SCHD)	7	Minnesota Multiphasic Personality Inventory (MMPI) items that as pooled best detect schizophrenia	Golden and Meehl, [25]
Symptom Check-List, Depression subscale (SCL-D)	13	Used as a screen for depression in normal population	Derogatis et al., [6]; Fink et al., [17]
Temperament and Character Inventory (TCI) subscales:			
(i) novelty seeking (TCI-NS)	40	Respond with intense excitement to novel stimuli	Cloninger et al., [7]
(ii) harm avoidance (TCI-HA)	35	Subjects with tendency to respond intensively to signals of aversive stimuli, thereby inhibiting/stopping behaviour	Cloninger et al., [7]
(iii) reward dependence (TCI-RD)	24	Respond intensely to signals of reward	Cloninger et al., [7]
(iv) persistence (TCI-P)	8	Subjects with tendency to persevere in behaviours associated with reward	Cloninger et al., [7]

**Table 2:** Mean and standard deviation (SD) of psychological scales in the 31-year followup (in 1997) for those with self-report or previous hospitalisation due to depression, other psychiatric disorders, and for those without psychiatric disorders.

	(A) Depression until 1997			(B) Other psychiatric disorders			(C) No psychiatric disorders			Effect sizes (Cohen's *d*)		
				until 1997			until 1997			(A) versus (B)	(A) versus (C)	(B) versus (C)
	*N*	Mean	SD	*N*	Mean	SD	*N*	Mean	SD	*F* (*d*)	*F* (*d*)	*F* (*d*)
Psychopathology scales:												
Physical Anhedonia Scale (PAS)	202	14.68	8.19	149	16.34	7.86	4572	15.02	6.97	0.74 (−0.21)	0.49 (−0.05)	5.24^∗^ (0.19)
Social Anhedonia Scale (SAS)	200	11.60	6.78	146	11.99	6.75	4569	9.33	5.43	0.00 (−0.06)	48.43^∗∗∗^ (0.41)	31.54^∗∗∗^ (0.49)
Perceptual Aberration Scale (PER)	202	5.09	5.64	147	4.29	5.85	4568	2.24	3.04	3.86 (0.14)	149.00^∗∗∗^ (0.90)	34.05^∗∗∗^ (0.65)
Hypomanic Personality Scale (HPS)	201	15.11	7.44	147	15.07	8.78	4571	11.07	6.92	0.26 (0.01)	60.89^∗∗∗^ (0.58)	29.44^∗∗∗^ (0.57)
Bipolar II Scale (BIP2)	202	12.99	4.46	148	12.91	4.39	4577	10.47	3.73	0.57 (0.02)	88.75^∗∗∗^ (0.67)	42.63^∗∗∗^ (0.65)
Schizoidia Scale (SCHD)	202	3.51	1.61	147	3.31	1.56	4578	2.51	1.39	1.21 (0.13)	89.90^∗∗∗^ (0.72)	39.89^∗∗∗^ (0.57)
SCL-Depression (SCL-D)	202	1.78	0.58	144	1.66	0.56	4571	1.33	0.33	4.54^∗^ (0.20)	310.90^∗∗∗^ (1.29)	92.19^∗∗∗^ (0.97)
Temperament dimensions:												
novelty seeking (TCI-NS)	200	21.29	5.82	145	21.84	6.13	4534	20.34	5.86	0.83 (−0.09)	3.62 (0.16)	6.91^∗∗^ (0.26)
harm avoidance (TCI-HA)	200	18.09	7.36	145	17.21	6.64	4534	13.82	6.00	0.94 (0.12)	86.49^∗∗∗^ (0.70)	42.03^∗∗∗^ (0.56)
reward dependence (TCI-RD)	200	14.95	3.95	145	14.30	3.89	4534	14.74	3.78	0.23 (0.16)	0.31 (0.05)	1.40 (−0.12)
persistence (TCI-P)	200	4.28	1.86	145	4.09	1.89	4534	4.31	1.74	0.66 (0.10)	0.02 (−0.02)	2.76 (−0.12)

TCI: Temperament and Character Inventory. Mean values in categories are compared using two-way analysis of variance (ANOVA), with diagnostic group and gender as independent variables. Statistical significant effect sizes are marked with asterisks as follows: ^∗^ *P* < 0.05, ^∗∗^ *P* < 0.01, and ^∗∗∗^ *P* < 0.001.

**Table 3:** Mean and standard deviation (SD) of psychological scales in the 31-year followup (in 1997) for those with hospitalisation of depression or other psychiatric disorders and for those without hospitalization due to psychiatric disorders in 1998–2010.

	(A) Depression in 1998–2010			(B) Other psychiatric disorders			(C) No psychiatric disorders			Effect sizes (Cohen's *d*)		
				in 1998–2010			until 2010			(A) versus (B)	(A) versus (C)	(B) versus (C)
	*N*	Mean	SD	*N*	Mean	SD	*N*	Mean	SD	*F* (*d*)	*F* (*d*)	*F* (*d*)
Psychopathology scales:												
Physical Anhedonia Scale (PAS)	51	15.88	6.08	57	18.07	7.24	4464	14.97	6.97	1.77 (−0.33)	0.43 (0.13)	6.16^∗^ (0.44)
Social Anhedonia Scale (SAS)	51	11.69	5.82	57	11.98	6.78	4461	9.27	5.39	0.00 (−0.05)	9.37^∗∗^ (0.45)	9.75^∗∗^ (0.50)
Perceptual Aberration Scale (PER)	51	2.96	2.75	57	2.95	3.45	4460	2.22	3.04	0.04 (0.00)	3.30 (0.24)	4.12^∗^ (0.24)
Hypomanic Personality Scale (HPS)	51	12.37	6.65	57	12.32	8.19	4463	11.04	6.91	0.00 (0.01)	2.10 (0.19)	2.62 (0.18)
Bipolar II Scale (BIP2)	51	12.37	3.56	57	11.68	4.50	4469	10.44	3.71	0.81 (0.17)	13.49^∗∗∗^ (0.52)	5.75^∗^ (0.33)
Schizoidia Scale (SCHD)	51	3.14	1.66	57	2.65	1.62	4470	2.50	1.38	1.97 (0.30)	12.09^∗∗∗^ (0.46)	1.77 (0.11)
SCL-Depression (SCL-D)	51	1.49	0.46	55	1.45	0.41	4465	1.33	0.33	2.38 (0.09)	12.46^∗∗∗^ (0.48)	9.22^∗∗^ (0.36)
Temperament dimensions:												
novelty seeking (TCI-NS)	51	19.78	6.20	57	21.68	5.75	4426	20.33	5.86	3.30 (−0.32)	0.33 (−0.09)	4.08^∗^ (0.23)
harm avoidance (TCI-HA)	51	16.96	6.28	57	15.02	7.68	4426	13.77	5.96	1.44 (0.28)	16.11^∗∗∗^ (0.54)	4.29^∗^ (0.21)
reward dependence (TCI-RD)	51	14.24	3.99	57	13.68	3.51	4426	14.76	3.78	0.22 (0.15)	0.52 (−0.14)	1.46 (−0.29)
persistence (TCI-P)	51	4.53	1.76	57	4.07	1.93	4426	4.31	1.73	1.86 (0.25)	0.65 (0.13)	1.91 (−0.14)

TCI: Temperament and Character Inventory. Mean values in categories are compared to using two-way analysis of variance (ANOVA), with diagnostic group and gender as independent variables. Statistical significant effect sizes are marked with asterisks as follows: *P < 0.05, ^∗∗^ *P* < 0.01, and ^∗∗∗^ *P* < 0.001.

## References

[1] Maruish ME (1999). *The Use of Psychological Testing for Treatment Planning and Outcomes Assessment*.

[2] American Psychiatric Association (2000). *Handbook of Psychiatric Measures*.

[3] Gottesman II, Gould TD (2003). The endophenotype concept in psychiatry: etymology and strategic intentions. *American Journal of Psychiatry*.

[4] Hasler G, Drevets WC, Manji HK, Charney DS (2004). Discovering endophenotypes for major depression. *Neuropsychopharmacology*.

[5] Bearden CE, Freimer NB (2006). Endophenotypes for psychiatric disorders: ready for primetime?. *Trends in Genetics*.

[6] Derogatis LR, Lipman RS, Covi L (1973). SCL-90: an outpatient psychiatric rating scale—preliminary report. *Psychopharmacology Bulletin*.

[7] Cloninger CR, Svrakic DM, Przybeck TR (1993). A psychobiological model of temperament and character. *Archives of General Psychiatry*.

[8] Miettunen J, Raevuori A (2011). A meta-analysis of temperament in axis I psychiatric disorders. *Comprehensive Psychiatry*.

[9] Cloninger CR, Svrakic DM, Przybeck TR (2006). Can personality assessment predict future depression? A twelve-month follow-up of 631 subjects. *Journal of Affective Disorders*.

[10] Farmer RF, Seeley JR (2009). Temperament and character predictors of depressed mood over a 4-year interval. *Depression and Anxiety*.

[11] Loas G, Boyer P, Legrand A (1999). Anhedonia in the deficit syndrome of schizophrenia. *Psychopathology*.

[12] Rantakallio P (1969). Groups at risk in low birth weight infants and perinatal mortality. *Acta Paediatrica Scandinavica*.

[13] Isohanni M, Mäkikyrö T, Moring J (1997). A comparison of clinical and research DSM-III-R diagnoses of schizophrenia in a Finnish national birth cohort. Clinical and research diagnoses of schizophrenia. *Social Psychiatry and Psychiatric Epidemiology*.

[14] Moilanen K, Veijola J, Läksy K (2003). Reasons for the diagnostic discordance between clinicians and researchers in schizophrenia in Northern Finland 1966 Birth Cohort. *Social Psychiatry and Psychiatric Epidemiology*.

[15] Haapea M, Miettunen J, Läärä E (2008). Non-participation in a field survey with respect to psychiatric disorders. *Scandinavian Journal of Public Health*.

[16] Chapman LJ, Chapman JP (1986). *Infrequency Scale for Personality Measures*.

[17] Fink P, Jensen J, Borgquist L (1995). Psychiatric morbidity in primary public health care: a Nordic multicentre investigation Part I: method and prevalence of psychiatric morbidity. *Acta Psychiatrica Scandinavica*.

[18] Veijola J, Jokelainen J, Läksy K (2003). The Hopkins Symptom Checklist-25 in screening DSM-III-R axis-I disorders. *Nordic Journal of Psychiatry*.

[19] American Psychiatric Association (1987). *DSM-III-R: Diagnostic and Statistical Manual of Mental Disorders*.

[20] Chapman LJ, Chapman JP, Raulin ML (1976). Scales for physical and social anhedonia. *Journal of Abnormal Psychology*.

[21] Eckblad M, Chapman LJ, Chapman JP, Mishlove M (1982). *The Revised Social Anhedonia Scale*.

[22] Chapman LJ, Chapman JP, Raulin ML (1978). Body-image aberration in schizophrenia. *Journal of Abnormal Psychology*.

[23] Eckblad M, Chapman LJ (1986). Development and validation of a scale for hypomanic personality. *Journal of Abnormal Psychology*.

[24] Akiskal HS, Maser JD, Zeller PJ (1995). Switching from “unipolar” to bipolar II: an 11-year prospective study of clinical and temperamental predictors in 559 patients. *Archives of General Psychiatry*.

[25] Golden RR, Meehl PE (1979). Detection of the schizoid taxon with MMPI indicators. *Journal of Abnormal Psychology*.

[26] Miettunen J, Kantojärvi L, Ekelund J (2004). A large population cohort provides normative data for investigation of temperament. *Acta Psychiatrica Scandinavica*.

[27] Miettunen J, Veijola J, Freimer N (2010). Data on schizotypy and affective scales are gender and education dependent—study in the Northern Finland 1966 Birth Cohort. *Psychiatry Research*.

[28] Miettunen J, Veijola J, Lauronen E, Kantojärvi L, Joukamaa M (2007). Sex differences in Cloninger’s temperament dimensions-a meta-analysis. *Comprehensive Psychiatry*.

[29] Miettunen J, Jääskeläinen E (2010). Sex differences in wisconsin schizotypy scales-a meta-analysis. *Schizophrenia Bulletin*.

[30] Cohen J (1992). A power primer. *Psychological Bulletin*.

[31] Loas G (1995). Anhedonia in major depressive disorders. A comparative study between 55 major depressives (RDC) and 54 healthy subjects. *Encephale*.

[32] Berlin I, Givry-Steiner L, Lecrubier Y, Puech AJ (1998). Measures of anhedonia and hedonic responses to sucrose in depressive and schizophrenic patients in comparison with healthy subjects. *European Psychiatry*.

[33] Katsanis J, Iacono WG, Beiser M (1990). Anhedonia and perceptual aberration in first-episode psychotic patients and their relatives. *Journal of Abnormal Psychology*.

[34] Blanchard JJ, Horan WP, Brown SA (2001). Diagnostic differences in social anhedonia: a longitudinal study of schizophrenia and major depressive disorder. *Journal of Abnormal Psychology*.

[35] Loas G, Salinas E, Guelfi JD, Samuel-Lajeunesse B (1992). Physical anhedonia in major depressive disorder. *Journal of Affective Disorders*.

[36] Miettunen J, Veijola J, Isohanni M (2011). Identifying schizophrenia and other psychoses with psychological scales in the generasl population. *Journal of Nervous and Mental Disease*.

[37] Sandanger I, Moum T, Ingebrigtsen G, Dalgard OS, Sørensen T, Bruusgaard D (1998). Concordance between symptom screening and diagnostic procedure: the Hopkins Symptom Checklist-25 and the Composite International Diagnostic Interview I. *Social Psychiatry and Psychiatric Epidemiology*.

[38] Abrams KY, Yune SK, Kim SJ (2004). Trait and state aspects of harm avoidance and its implication for treatment in major depressive disorder, dysthymic disorder, and depressive personality disorder. *Psychiatry and Clinical Neurosciences*.

[39] Kampman O, Poutanen O (2011). Can onset and recovery in depression be predicted by temperament? A systematic review and meta-analysis. *Journal of Affective Disorders*.

[40] de Winter RFP, Wolterbeek R, Spinhoven P, Zitman FG, Goekoop JG (2007). Character and temperament in major depressive disorder and a highly anxious-retarded subtype derived from melancholia. *Comprehensive Psychiatry*.

[41] Lehtinen V, Joukamaa M (1994). Epidemiology of depression: prevalence, risk factors and treatment situation. *Acta Psychiatrica Scandinavica, Supplement*.

